# Artificial neural networks versus proportional hazards Cox models to predict 45-year all-cause mortality in the Italian Rural Areas of the Seven Countries Study

**DOI:** 10.1186/1471-2288-12-100

**Published:** 2012-07-23

**Authors:** Paolo Emilio Puddu, Alessandro Menotti

**Affiliations:** 1 Laboratory of Biotechnologies Applied to Cardiovascular Medicine, Department of Cardiovascular, Respiratory, Nephrological, Anesthesiological and Geriatrical Sciences, Sapienza, University of Rome, Viale del Policlinico, 155, Rome 00161, Italy; 2 Associazione per la Ricerca Cardiologica, Rome, Italy

**Keywords:** Neural networks, Cox models, Prediction, All-cause mortality, 45-year follow-up, Epidemiology, Seven countries study

## Abstract

****Background**:**

Projection pursuit regression, multilayer feed-forward networks, multivariate adaptive regression splines and trees (including survival trees) have challenged classic multivariable models such as the multiple logistic function, the proportional hazards life table Cox model (Cox), the Poisson’s model, and the Weibull’s life table model to perform multivariable predictions. However, only artificial neural networks (NN) have become popular in medical applications.

****Results**:**

We compared several Cox versus NN models in predicting 45-year all-cause mortality (45-ACM) by 18 risk factors selected *a priori:* age; father life status; mother life status; family history of cardiovascular diseases; job-related physical activity; cigarette smoking; body mass index (linear and quadratic terms); arm circumference; mean blood pressure; heart rate; forced expiratory volume; serum cholesterol; corneal arcus; diagnoses of cardiovascular diseases, cancer and diabetes; minor ECG abnormalities at rest. Two Italian rural cohorts of the Seven Countries Study, made up of men aged 40 to 59 years, enrolled and first examined in 1960 in Italy. Cox models were estimated by: a) forcing all factors; b) a forward-; and c) a backward-stepwise procedure. Observed cases of deaths and of survivors were computed in decile classes of estimated risk. Forced and stepwise NN were run and compared by C-statistics (ROC analysis) with the Cox models. Out of 1591 men, 1447 died. Model global accuracies were extremely high by all methods (ROCs > 0.810) but there was no clear-cut superiority of any model to predict 45-ACM. The highest ROCs (> 0.838) were observed by NN. There were inter-model variations to select predictive covariates: whereas all models concurred to define the role of 10 covariates (mainly cardiovascular risk factors), family history, heart rate and minor ECG abnormalities were not contributors by Cox models but were so by forced NN. Forced expiratory volume and arm circumference (two protectors), were not selected by stepwise NN but were so by the Cox models.

****Conclusions**:**

There were similar global accuracies of NN versus Cox models to predict 45-ACM. NN detected specific predictive covariates having a common thread with physical fitness as related to job physical activity such as arm circumference and forced expiratory volume. Future attention should be concentrated on why NN versus Cox models detect different predictors.

## Background

The predictive power assessment of risk factors by multivariable models such as the multiple logistic function, the proportional hazards life table Cox model, the Poisson’s model, and the Weibull’s life table model, is one of the cogent problems of contemporary cardiovascular epidemiology since the selection among these standard methods [1-3] has been challenged by other methods to perform multivariable predictions. New models included projection pursuit regression [4], multilayer feed-forward networks [5] and multivariate adaptive regression splines [6]. Other methods, particularly trees (including survival trees) have been employed [7-10], although back prop nets may well perform better. However, among new comers [4-12] only artificial neural networks have become popular in medical applications [11,12], which could also be possibly due to a lot of attention being focused on these techniques in other fields and so have become known in medicine. This larger acceptance and applicability relates to widespread availability of free-, share-, and commercial-ware [see: http://neuralnetworks.ai-depot.com/Software.html and the recent increase of personal computer power. On the other hand, the necessity has been felt to cope with the limitations of methods such as logistic regression [13]. In fact, receiver operating characteristic (ROC) curves, indexing global predictive accuracy of logistic models [14-17] also comparatively [18-20], rarely exceeded 0.75 in the majority of epidemiological or clinical cardiovascular investigations [21,22].

### Implementation

When the performance and/or reliability of predictive models is limited, or of low sensitivity and specificity, their capability may be hampered to identify high risk subjects who deserve individualized treatment [21]. The neural network method stems [11,12,23,24] from its potential for improved predictive performance by exploring hidden layers to find nonlinearities, interactions and nonlinear interactions among predictors, particularly when the data are continuous [25]. The attraction of neural networks is quite evident from the impressive growth of results published with these methods in the last 20 years [12]. However, there are relatively few comparative reports on the performance and accuracy of neural networks, which were assessed only versus multiple logistic function, to predict events in clinical [26,27] or epidemiological [28,29] cardiovascular studies and none has been performed versus models taking time into account such as the proportional hazards Cox model.

For the purpose of the present investigation we selected *a priori*[30] a series of covariates among those previously studied [31] to assess 40-year all-cause mortality predictive power among middle-aged men of the Italian Rural Areas (IRA) of the Seven Countries Study (SCS). We used the 45-year survival data to compare the global predictive accuracy of Cox and neural network models.

### Cohorts and risk factors

The epidemiological material used for this analysis derives from the two Italian rural cohorts of the Seven Countries Study of Cardiovascular Diseases, made up of men aged 40 to 59 years, enrolled and first examined in 1960 [32] using standard methods [33,34]. They represented 98.8% (n = 1712) of defined samples belonging to the rural communities of Crevalcore in Northern Italy and Montegiorgio in Central Italy. For the purposes of this analysis only baseline measurements of risk factors were considered, together with information on mortality over 45 years, although several re-examinations were conducted after the entry one.

Risk factors used in this analysis were those identified as significant in a previous analysis dealing with 40 years of follow-up of all cause mortality (except xantelasma, too rare and unstable) [31], plus heart rate, minor ECG abnormalities and family history of cardiovascular diseases which were promising but not reaching significance in the previous analysis. Altogether they were the following: age; father life status; mother life status; family history of cardiovascular diseases; job-related physical activity; cigarette smoking; body mass index (linear and quadratic terms); arm circumference; mean blood pressure; heart rate; forced expiratory volume; serum cholesterol; corneal arcus; diagnoses of cardiovascular diseases, cancer and diabetes; minor ECG abnormalities at rest. Unit of measurements and technical details, are reported in Additional file 1: Appendix 1.

Collection of data on vital status and causes of death was complete for 45 years. Causes of death were coded but not used for this analysis. The baseline survey was conducted well before the era of the Helsinki Declaration. On the occasion of subsequent examinations, verbal consent was obtained in view of collecting follow-up data. The end-point of this analysis was all-cause mortality in 45 years, and the corresponding survival in some analyses. The analysis was conducted on 1591 men who had all the measurements available.

### Statistical analysis

Data are expressed as means ± SD or proportions and SE (when appropriate). Follow-up data, during 45 years, were investigated by modelling the presence (coded 1) or absence (coded 0) of all-cause mortality using the proportional hazards model [31]. Cox proportional hazards models were estimated by: a) forcing all factors; b) a forward stepwise procedure; and c) a backward stepwise procedure (with p = < 0.05 as selection criterion for stepwise procedures). Plots of Schönfeld residuals over time were produced to test the proportionality of hazard. The coefficients and constants of the models were applied back to the original risk factor levels of all men, to obtain an estimated risk of death. Observed cases of survivors were computed in decile classes of estimated risk. NCSS software version 2007 (released August 14, 2007 by J Hintze, Kaysville, Utah; see http://www.ncss.com) was used.

Tiberius Data Mining © software (version 5.4.3; see http://www.tiberius.biz) was used to obtain multilayer perceptron (MLP) neural network solutions (see Additional file 1: Appendix 2 for details). Briefly, these were from a 3-layer network, including the hidden unit containing 2 neurons (one linear and the second nonlinear), with 18 input nodes (corresponding to the 18 risk factors selected for Cox model) and one output unit, modelling the dichotomous risk outcome (for details and examples see Additional file 1: Appendix 2). MLPs were trained on all patterns but preventing over-fitting [12], using procedures substantially similar to the forced or forward stepwise methods used by the Cox model. A method similar to bootstrap was used on 10 consecutive runs to obtain MPLs by both forced and forward methods. Corrado Gini’s coefficient and graph [35] were produced. A Gini coefficient is the area under the diagonal and the curve whereas the area under the curve is the total area under an ROC. Therefore it is easy to obtain: ROC = (Gini*0.5) + 0.5. MedCalc software (version 9.6.3.0; see http://www.medcalcsoftware.com) was used to calculate the area under an ROC with 95% confidence intervals (CI) and make comparisons [14,15,19]. ROCs were compared between models and among solutions obtained. A value of p<0.05 was considered statistically significant in all cases.

## Results

Baseline characteristics are presented in Additional file 1: Appendix 1. Out of 1591 men entering the analysis 1447 died in 45 years (91%). Table 1 shows the results of 3 proportional hazards models to predict 45-year all cause mortality by either forcing all variables or by forward or backward stepwise approaches. The β coefficients and t values are shown along with hazard ratios and their 95% confidence intervals. In the forced Cox’ model, out of 18 pre-selected variables there were 13 covariates significantly associated with all-cause mortality. A direct relation was present for 10 covariates (age, father and mother life status, corneal arcus, cigarettes smoked per day, mean blood pressure, serum cholesterol and the prevalences of cardiovascular disease, cancer and diabetes), an inverse relation for one covariate (forced expiratory volume), whereas body mass index showed an inverse J shaped relation. Global accuracy by forced Cox model was extremely high (ROC > 0.810). Global accuracy (by ROC statistics) was not significantly different by adopting a stepwise approach (either forward or backward) and the covariates were substantially the same (with similar β coefficients) as compared to those selected by the forced approach. There was however an exception: arm circumference was selected (with an inverse relation) by forward stepwise Cox model, whereas backward stepwise Cox model selected instead physical activity (also with an inverse relation) pointing to physical fitness as the common descriptor. In fact, in the forced model both variables, although not statistically significant there, had an inverse relation with all-cause mortality. On the other hand, family history of CVD, heart rate and minor ECG abnormalities were not contributors.

**Table 1:** Proportional hazards models predicting 45-year all-cause mortality (1447 deaths among 1591 men) by three methods

	**Forced Cox model**			**Forward stepwise Cox model**			**Backward stepwise Cox model**		
	**(N = 1591)**			**(N = 1591)**			**(N = 1591)**		
	**β**	**t**	**HR(±95%CI)**	**β**	**t**	**HR(±95%CI)**	**β**	**t**	**HR(±95%CI)**
Age (years)	0.1024	16.81	1.68(1.58-1.79)	0.1017	16.78	1.67(1.58-1.78)	0.1029	17.01	1.69(1.59-1.79)
Father status (codes 0–1)	0.1422	2.19	1.15(1.01-1.31)	0.1421	2.19	1.15(1.01-1.31)	0.1436	2.21	1.15(1.02-1.31)
Mother status (codes 0–1)	0.2063	3.11	1.23(1.08-1.40)	0.2121	3.22	1.24(1.09-1.41)	0.2072	3.16	1.23(1.08-1.40)
Family history of CVD (codes 0–1)	0.0326	0.60	1.03(0.93-1.15)	=	=	=	=	=	=
Corneal arcus (codes 0–1)	0.2092	2.70	1.23(1.06-1.43)	0.1988	2.57	1.22(1.05-1.42)	0.2037	2.63	1.23(1.05-1.43)
Physical activity (codes 1-2-3)	−0.0830	−1.92	0.92(0.85-1.00)	=	=	=	−0.1042	−2.51	0.90(0.83-0.98)
Cigarettes smoked per day (N)	0.0174	6.26	1.18(1.12-1.24)	0.0177	6.37	1.18(1.12-1.25)	0.0177	6.36	1.18(1.12-1.25)
Body mass index (Kg/m^2^)	−0.1840	−2.77	0.51(0.31-0.82)	−0.1711	−2.62	0.53(0.33-0.85)	−0.2097	−3.30	0.46(0.29-0.73)
Body mass index^2^ [(Kg/m^2^)^2^]	0.0033	2.70	1.90(1.19-3.04)	0.0031	2.62	1.85(1.17-2.93)	0.0036	3.03	2.02(1.28-3.18)
Arm circumference (cm)	−0.0026	−1.64	0.94(0.88-1.01)	−0.0034	−2.21	0.92(0.86-0.99)	=	=	=
Mean blood pressure (mmHg)	0.0180	7.76	1.28(1.20-1.36)	0.0186	8.24	1.29(1.21-1.36)	0.0189	8.43	1.29(1.22-1.37)
Heart rate (beats/min)	0.0018	0.78	1.02(0.97-1.09)	=	=	=	=	=	=
Serum cholesterol (mmol/l)	0.0722	2.81	1.08(1.02-1.14)	0.0758	2.97	1.08(1.03-1.14)	0.0751	2.94	1.08(1.03-1.14)
Forced expiratory volume (l/m^2^)	−0.4691	−3.93	0.89(0.84-0.94)	−0.4815	−4.05	0.89(0.84-0.94)	−0.4742	−3.99	0.89(0.84-0.94)
ECGm abnormalities (codes 0–1)	0.0518	0.47	1.05(0.85-1.31)	=	=	=	=	=	=
Prevalence CVD (codes 0–1)	0.3658	2.85	1.44(1.12-1.85)	0.3520	2.75	1.42(1.11-1.83)	0.3905	3.05	1.48(1.15-1.90)
Prevalence K (codes 0–1)	2.1479	4.72	8.57(3.51-20.89)	2.2056	4.87	9.08(3.73-22.07)	2.1644	4.76	8.71(3.58-21.22)
Prevalence DIAB (codes 0–1)	0.2545	2.06	1.29(1.01-1.64)	0.2502	2.02	1.28(1.01-1.64)	0.2674	2.16	1.31(1.03-1.66)
									
ROC ± standard error (±95%CI)		0.829 ± 0.0137 (0.810-0.847)			0.830 ± 0.0136 (0.811-0.849)			0.830 ± 0.0137 (0.810-0.848)		

*β* = coefficient; *t* = t value of coefficient (when t > |1.96| p<0.05); *HR* = hazard ratio; *CI* = confidence intervals. The differences for hazards ratio are expressed as 0–1 for dichotomic variables, as 1 unit for physical activity, and as standard deviations for continuous variables.

*CVD* = cardiovascular disease; *DIAB* = diabetes; *ECGm* = minor ECG; *K* = cancer.

**Figure 1:**
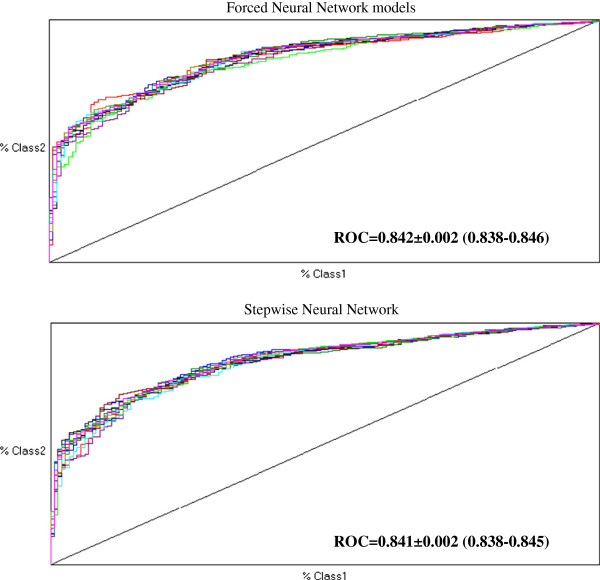
**Receiver operating characteristic curve by forced and stepwise neural network models obtained by 10 runs.** ROC = receiver operating characteristic curve (95% confidence intervals).

**Figure 2:**
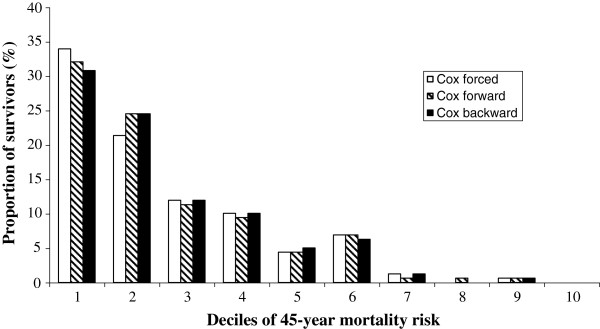
Proportion of survivors in 45 years distributed in decile classes of estimated risk of death according to Cox models.

Table 2 shows the results of forced and stepwise multiplayer perceptron models to predict 45-year all-cause mortality in IRA SCS cohorts whose multiple-run results are illustrated in Figure 1 along with global accuracies. By inspecting Gini coefficients produced by Tiberius software the incremental contribution of each one among the 18 covariates might be appreciated. By forced neural network there were 16 contributing covariates whereas by stepwise neural model there were 13 covariates contributing to 45-year all cause prediction. Different from Cox models, family history, heart rate and minor ECG abnormalities were selected among contributors, whereas physical activity and prevalence of cancer were not. Moreover, whereas forced neural network selected body mass index (both indices), arm circumference and forced expiratory volume, stepwise neural network did not. Notwithstanding these differences Table 3 indicates no statistical differences between couple-comparisons of ROCs among Cox versus neural network models. On the other hand, the distribution of survivors in decile classes of 45-year estimated mortality risk were substantially similar when assessed by each one of the 3 Cox models, with large differences across deciles (Figure 2).

**Table 2:** Multilayer perceptron models predicting 45-year all-cause mortality (1447 deaths among 1591 men) by two methods

**Rank**	**Gini**	**Variable**	**Keep**
**NEURAL NETWORK FORCED**			
1	0.15950	Age (AGE0: years)	1
2	0.60615	Cigarettes smoked per day (CIG0: N)	1
3	0.64475	Family history of CVD(famcv0: codes 0–1)	1
4	0.64790	Mean blood pressure (MBP: mmHg)	1
5	0.65872	Serum cholesterol (CHOL0: mmol/l)	1
6	0.67052	Corneal arcus (gero0: codes 0–1)	1
7	0.67484	Mother status (Mother0: codes 0–1)	1
8	0.67680	Prevalence DIAB (pdiab0: codes 0–1)	1
9	0.68081	Forced expiratory volume (fev0trans0: l/m^2^)	1
10	0.68321	Heart rate (hr0: beats/min)	1
11	0.68362	Father status (Father0: codes 0–1)	1
12	0.68414	Body mass index (BMI0: Kg/m^2^)	1
13	0.68516	Minor ECG abnormalities (MinorECG0: codes 0–1)	1
14	0.68524	Prevalence CVD (Pcvd0: codes 0–1)	1
15	0.68579	Arm circumference (midclean0: cm)	1
16	0.68664	Body mass index^2^ (BMIsq0) [(Kg/m^2^)^2^]	1
17	0.68813	Prevalence cancer (pcan0: codes 0–1)	0
18	0.68872	Physical activity (PHYAC0: codes 1-2-3)	0
**NEURAL NETWORK STEPWISE**			
1	0.17824	Age (years)	1
2	0.60661	Cigarettes smoked per day (N)	1
3	0.65370	Mean blood pressure (mmHg)	1
4	0.67844	Mother status (codes 0–1)	1
5	0.67918	Corneal arcus (codes 0–1)	1
6	0.68104	Heart rate (beats/min)	1
7	0.68347	Father status (codes 0–1)	1
8	0.68468	Minor ECG abnormalities (codes 0–1)	1
9	0.69231	Physical activity (codes 1-2-3)	1
10	0.69329	Family history (codes 0–1)	1
11	0.69480	Prevalence DIAB (codes 0–1)	1
12	0.69603	Serum cholesterol (mmol/l)	1
13	0.69854	Prevalence CVD (codes 0–1)	1

**Table 3:** Comparisons among receiver operating characteristics curves obtained by Cox versus neural network models predicting 45-year all-cause mortality (1447 deaths among 1591 men)

**First Model**	**Compared to the Model**	**p**
Cox forced	Cox forward stepwise	0.9587
Cox forced	Cox backward stepwise	0.9588
Cox forced	Neural network forced	0.3478
Cox forced	Neural network stepwise	0.3681
Cox forward stepwise	Cox backward stepwise	1.0000
Cox forward stepwise	Neural network forced	0.3478
Cox forward stepwise	Neural network stepwise	0.4236
Cox backward stepwise	Neural network forced	0.3861
Cox backward stepwise	Neural network stepwise	0.4269
Neural network forced	Neural network stepwise	0.7237

## Discussion

This is the first investigation to have ever compared in epidemiological material several methods to run Cox versus neural network models to predict 45-year all-cause mortality by a set of 18 risk factors (of which half were continuous) selected *a priori.* The global accuracies, assessed by C-statistics (ROC analysis) were extremely high by all methods but there was no clear-cut superiority of any model to predict 45-year all-cause mortality. There are inter-model variations to select predictive covariates among baseline variables. In particular, whereas all models concurred to define the role of 10 covariates (age, father and mother status, corneal arcus, cigarettes smoked per day, mean blood pressure, serum cholesterol and the prevalences of cardiovascular disease, cancer and diabetes), family history of CVD, heart rate and minor ECG abnormalities were not contributors by Cox models but were so by forced neural network model. Special attention needs be directed to the protective roles of forced expiratory volume and arm circumference, which may have a common thread with physical fitness as related to job physical activity [31,32], since these variables were not selected by stepwise neural network (selecting instead physical activity) but were so by Cox models. Finally, the overall picture indicates an inverse J shaped relations for body mass index (except than by stepwise neural network).

### Multivariable statistics and neural networks

There are excellent recent books [1,2,17] to have covered proportional hazards life table Cox model [36] and its use to assess the relationship between covariates and events including mortality. On the other hand, multilayer feed-forward networks were demonstrated by Hornik et al. with appropriate internal parameters (weights) to approximate an arbitrary non-linear function [5]. Because prediction can be restated as a function approximation problem, it follows that artificial neural networks have the potential to solve major problems in a wide range of applications where their use has been reviewed to show advantages and disadvantages for predicting medical outcomes [12]. What is particularly important with neural networks is that a multi-factorial function can be fitted in such a way that creating the functional form and fitting the function are performed at the same time, unlike non-linear regression in which a fit is forced to a pre-chosen function. This capability gives neural networks, at least potentially, an advantage over traditional statistical multivariable regression techniques [12].

Dayhoff and De Leo have recently reviewed what is inside the black box of neural network models in describing the most popular squashing function (also known as activation function) by which multilayered perceptron actually operates (see Additional file 1: Appendix 2). They have pointed out that with neural networks it is possible to mediate predictions for individual patients with prevalence and misclassification cost considerations using ROC methodology [12]. When a neural network is trained on a compendium of data, it builds a predictive model based on those data, by reflecting a minimization in error when the network’s prediction (its output) is compared with a known or expected outcome. Performance measurements would be taken to report the neural network’s level of success. The trained neural network then can be used to classify each new individual subject. This represents “a paradigm shift”, compared to previous methods whereby statistics concerning given populations are computed and published and a new individual subject then is referenced to the closest matching patient population for clinical decision support [12].

With all modelling methods an important part is the selection (and the number) of prognostic variables to be included in the model. The selection may be done *a priori* based on previous knowledge, as it was done in the present investigation, to prevent the data driven method used more often than not, which leads to a different set of variables being selected each time [30]. However, some inspiration was taken by a previous experience on this material exploring 40-year predictive capabilities of all-cause mortality [31]. The results of our study also showed that it is indeed important to take into consideration also the methods used to run the predictive models. In fact, when several variables are included, one may not obtain directly comparable solutions among different studies (or here different models), if the selected procedure is stepwise. This reinforces the importance to have forced solutions with a full model considering all selected covariates.

### Comparing different predictive models

In the cardiovascular area, systematic comparisons of neural networks versus standard multivariable predictive models such as multiple logistic function has not been a common practice in either epidemiological [28,29] or clinical [21-27] investigations. Cox and neural networks were not previously compared. Voss et al. [28] analysed 10-year fatal (104 of 5.159 men, or 2%) and non-fatal (235 of 5.159 men, or 4.6%) CHD events among working men aged 35–65 years from the PROCAM study in Germany [28]. However, neural network and multiple logistic models were run with dissimilar covariates [30]. For example body mass index, height, presence and family history of hypertension were considered for neural network and not for multiple logistic function model. The conclusion was a superior performance of neural network versus logistic regression model in predicting 10-year CHD events. ROCs were 0.897 (95% CI 0.888-0.906) and 0.840 (95% CI 0.830-0.851), respectively [28].

Since the PROCAM experience lacked external validation of the neural network model [28], as commented by May [30], a necessary step to delineate not only its predictive accuracy and potency, but also its generalization, which might be a potential advantage over conventional regression techniques [11,12,37,38] we investigated 12763 men enrolled in the SCS. We compared 25-year CHD mortality and the predictive discrimination of the multilayer perceptron neural network versus multiple logistic function based on 4 standard, continuous risk factors, selected *a priori*. CHD mortality prediction by training neural network or multiple logistic function had similar ROCs (below 0.699). The external validation of neural network models derived from the high (USA) and low (Italy) risk populations yielded comparable ROCs similar to the logistic solutions in Northern and Eastern Europe, but higher ROCs in two areas [0.633 (logistic) vs. 0.665 or 0.666 (neural network: p<0.05) in Southern Europe and 0.676 (logistic) vs. 0.725 or 0.737 (neural network: p<0.01) in Japan]. Thus 25-year CHD prediction based on 4 continuous covariates showed lower global accuracies, both by neural network and logistic models [29], than 10-year CHD prediction based on 13 covariates [28].

### How to take advantage of all this

A lot of models in epidemiology, historically [32,39], have been linear-based on the principle of parsimony [1-3]. If the principle of linearity predicts the data well, why go to more complex models? The question has received replies from the continuous developments of theoretical mathematicians and the parallel increase of computer computation power, so that curvilinear models [13,36] are no more a problem also with personal computers [3,17,40-42]. This prompted new methods in the domain of survival prediction, among which neural networks [11,12,37,38] are only a few [4-10]. If neural networks are interesting, the interest should be in two aspects. First, the overall predictability of the model impact is at increasing the identification of high risk subjects who deserve individualized treatment [21]. The stakes are high as better models may lead to prevention of more deaths from CHD [30]. The second should be the actual pattern of prediction. Does the neural network give an answer which is intuitively more appealing and explanatory than the logistic regression or other models? This would be the most interesting aspect of the method [12]. With neural network methodology a meaningful prediction that is unique to each individual might be produced (see Additional file 1: Appendix 2). By applying ROC methodology to model outputs, the decision for individual subjects can be tailored further, since cost trade-off between false-positive and false-negative classifications might be examined [12].

There are obviously some limitations with neural networks but these may largely apply to standard multivariable methods as well. For example when applying neural networks for long-term (say more than 25 years) prediction of CHD deaths, the number of non-CHD deaths are too many and the time is not considered by neural networks. In this case, if non-CHD deaths are retained (as non cases) the predictive power of risk factors is diluted; if they are excluded the structure of the population and of its destiny are deformed. However, when prediction is referred to all-cause mortality as in the present study, these limitations do not operate. A further limitation of this analysis is bound to the use of a single measurement of personal characteristics employed for prediction, ignoring the time changes occurred along time. The satisfactory, and even more significant results separately obtained in the 40-year run of the IRA SCS cohort [31] made us confident in a valuable outcome of this analysis. The present essay may thus provide a baseline material to construct upon in the hope of further advancing our knowledge on risk factors for all-cause mortality prediction by neural networks.

### Consideration for the statistically-educated clinician

It is important to understand than global accuracy comparisons (using ROC curves) are often made on ‘hold-out’ data, eg data that was not used to generate models in the first place, so as to test the generality of the models. It is instead best to compare on new data, although this has been done quite rarely [29,43,44]. On the other hand, similar to the present results, several medical studies have found conventional regression techniques to perform as well or better than more complex techniques [27-29,44-46]. As for conventional techniques it is important to consider that full forced models should be used, since stepwise methods may convey unstable (or difficult to compare) results. As for more complex methods, an extremely important feature of the present study is the potential for a ‘paradigm shift’ in prediction, mentioned above in relation to neural network models. Although neural nets (and trees and various other machine learning techniques such as boosting, random forests etc.) allow individual predictions, back propagation neural network are still something of a ‘black box’ to the average clinician. Outputs of neural networks can be difficult to interpret for the ‘uninitiated’, although progress is being made.

### Long-term all-cause mortality prediction by risk factors

Comparisons with other studies are not easy due to different length of follow-up, different choice of risk factors and predictive models, and also because most of them dealt with a single risk factor, or few related risk factors. In the 30-year follow-up experience of the Framingham Study serum cholesterol was directly associated with all-cause mortality, at least for relatively younger subjects [47]: this was also observed here by all models, but was not the case in larger aggregate experience of the SCS in Europe [42]. All-cause mortality investigations were reviewed [42]. In the context of the General Post Office study in UK [48] among women and men aged 35–70, associations with systolic blood pressure were equally strong for women and men, that of serum cholesterol was higher in women, while associations with 2-hour glucose levels was observed only in men. The strongest, most consistent predictor of mortality was smoking in women and poor lung function in men. ECG ischemia, although associated with cardiovascular mortality in both sexes was not associated with all-cause mortality. In a Japanese study on elderly people, health behavior and social role were risk factors for all-cause mortality along with age, low serum albumin, high blood pressure and ECG abnormalities, among a total of 30 personal characteristics [49]. Other studies (always reviewed in [42]) considered single or very specific risk factors for all-cause mortality such as the limited influence of soil-cadmium levels, the null influence of radar equipment, the direct role of respiratory symptoms, alcohol abuse, left bundle branch block, post-load plasma glucose, high basal metabolic rates, high body mass index, pessimistic side of the Minnesota Multiphasic Personality Inventory Optimism-Pessimism Scale scores, and high physical work demand. None of previous investigations considered neural networks or undertook a comparative study of global predictive performance vs. Cox models or logistic regression.

## Conclusions

Following the external validation of neural networks [29], at least in the context of 25-year prediction of CHD mortality, a global conclusion should be that neural network models present some potential advantage [12], although the statistical difference as compared to standard multivariable methods such as logistic regression [28] or other more complex models [43] may have not a tremendous impact to call for their wider application. The evidence presented here about 45-year all-cause mortality prediction by 18 covariates is in line with these conclusions as ROCs were higher by neural networks (also in absolute terms since > 0.838: Figure 1) but there was no statistical differences vs. Cox models (ROCs > 0.810: Tables 1 and 3). A peculiarity may still reside on the capability of selecting covariates (such as in here arm circumference) that may go underestimated by different methods. Future attention should be concentrated on why neural network versus Cox models detect different predictors.

## Abbreviations

ACM, All-cause mortality; CHD, Coronary heart disease; IRA, Italian rural areas; ROC, Receiver operating characteristic; SCS, Seven countries study.

## Competing interests

The authors declare that they have no competing interests.

## Authors’ contributions

PEP and AM equally contributed to the design, analysis and writing of this manuscript. Both authors read and approved the final manuscript.

## Pre-publication history

The pre-publication history for this paper can be accessed here:

http://www.biomedcentral.com/1471-2288/12/100/prepub

## Supplementary Material

Additional file 1 **Appendix 1.** Risk factors measured at entry. Definitions, units of measurement, mean levels and use in analyses. **Appendix 2.** Neural network modelling. (DOC 105 kb) Click here for file
