# Supplementary material for: Artificial neural networks versus proportional hazards Cox models to predict 45-year all-cause mortality in the Italian Rural Areas of the Seven Countries Study
Source: BMC Med Res Methodol. 2012 Jul 23;12:100. doi: 10.1186/1471-2288-12-100 (PMC3549727; doi:10.1186/1471-2288-12-100)
Supplement: Additional file 1 — Appendix 1. Risk factors measured at entry. Definitions, units of measurement, mean levels and use in analyses. Appendix 2. Neural network modelling. (DOC 105 kb) [file 1471-2288-12-100-S1.doc]

**Appendix 1.** Risk factors measured at entry. Definitions, units of measurement, mean levels and use in analyses.

| **Risk factor**  **(abbreviation)** | | **Definition**  **or**  **Details** | | **Unit of measurement** | | **Mean and (SD)**  **or**  **Proportion % and (SE)** | |
| --- | --- | --- | --- | --- | --- | --- | --- |
|  | |  | |  | |  | |
| Age (AGE0) | | Approximated to the nearest birthday | | Years | | 49.7 (5.1) | |
|  | |  | |  | |  | |
| Father status  (Father0) | | Father died <65 years for non-infectious or non-violent causes | | 0 = no  1 = yes | | 20.9 % (1.02) | |
| Mother status  (Mother0) | | Mother died <65 years for non-infectious or non-violent causes | | 0 = no  1 = yes | | 20.5 % (1.01) | |
| Family history of cardiovascular diseases (famcv0) | | History of myocardial infarction, stroke or other defined cardiovascular diseases in 1^st^-degree siblings | | 0 = no  1 = yes | | 38.1 % (1.22) | |
|  | |  | |  | |  | |
| Physical activity  (PHYACA0) | | Job-related, derived from questions matched with reported occupation | | 1=sedentary  2=moderate  3=heavy | | 2.61 (0.65) | |
| Cigarette smoking  (CIG0) | | Current cigarette smoking derived from ad-hoc questionnaire | | N of cigarettes  per day on average | | 8.78 (9.52) | |
| Body mass index (BMI0) | | Weight / height squared | | kg / m^2^ | | 25.23 (3.68) | |
| Body mass  Index squared  (BMIsq0) | |  | | (Body mass index)^2^ | | 650.13 (196.47) | |
| Mid-arm circumference  (midclean0) | | Right arm. Method as from [29]  Mathematically cleaned from skin and subcutaneous tissue using the value of bicipital skinfold thickness | | Mm | | 269.07 ( 23.47) | |
| Mean blood pressure  (MBP) | | Supine.  Average of 2 measurements  Method as from [29].  Diastolic blood pressure + 1/3 of pulse pressure | | mm Hg | | 104.63 (13.52) | |
| Heart rate (hr0) | | From ECG, average rate in lead I and V_6_ | | beats /minute | | 71.02 (12.78) | |
| Minor ECG abnormalities  (MinorECG0) | | From Minnesota code, in absence of clinical diagnosis of heart disease.  Code 11 in [28] | | 0 = no  1 = yes | | 5.97 % (0.59) | |
|  | |  | |  | |  | |
| Forced expiratory volume  in ¾ sec (fev0trans0) | | Method as from [29].  Best of 2 tests.  Adjusted (divided) for height^2^ | | L / m^2^ | | 1.085 (0.25) | |
|  | |  | |  | |  | |
| Serum cholesterol  (CHOL0) | | Method of Abel-Kendal modified by Anderson and Keys [30]. Casual | | mmol / L | | 5.22 (1.06) | |
| Corneal arcus  (gero0) | | Clinical judgment | | 0 = no  1 = yes | | 13.8 % (0.87) | |
|  | |  | |  | |  | |
| Diagnosis  of any cardiovascular disease (Pcvd0) | | Any clinically overt cardiovascular disease by combining history, physical examination, ECG findings.  Seven Countries criteria [28] | | 0 = no  1 = yes | | 4.34 % (0.51) | |
| Diagnosis of cancer  (pcan0) | | Clinical judgment based on diagnosis and specific treatment | | 0 = no  1 = yes | | 0.31 % (0.14) | |
| Diagnosis of diabetes  (pdiab0) | | History of diabetes or use of anti-diabetic diet or specific drugs or glucose definitely present in urine | | 0 = no  1 = yes | | 4.78 % (0.53) | |
|  |  | |  | |  | |  |
|  |  | |  | |  | |  |

Abbreviations refer to names given in neural network modelling by Tiberius software.

SD = standard deviation; SE = standard error.

**Appendix 2.** Neural network modelling.

The relatively complex terminology adopted in the domain of artificial neural networks prompted some lay translations for the non-initiated to fully understand the approximate correspondence with more familiar terms used for traditional linear models such as the multiple logistic function and others [11,26]. Here is a list of comparative terms between these approaches [modified from 11]:

| Standard Multivariable Methods | Neural Networks |
| --- | --- |
| Independent (predictor) variable | Input |
| Dependent (outcome) variable | Output |
| Predicted probability | Transformed probability |
| Regression coefficient | Weight in the connection |
| Intercept parameter | Bias weight |
| Parameter estimation | Learning |
| Observation | Training case, pattern |

The most popular architecture currently adopted for artificial neural networks is the multilayered perceptron (MLP), which can be trained by back-propagation and typically is organized as a set of interconnected layers of artificial neurons. Each artificial neuron has an associated output activation level, which changes during the many computations that are performed during training. Each neuron receives inputs from multiple sources, and performs a weighted sum and a squashing function, the most popular being the sigmoid function, as follows:

F(x) = 1/1 + e(-x) (1)

in which x is the input to the squashing function and, in the neural network, is equal to S_j_ (node j), the sum of the products of the incoming activation levels with their associated weights, which is computed as follows:

S_j_ = Σ^n^ _i=0_ w_ji_ a_i_ (2)

in which w_ji_ is the incoming weight from unit i, a_i_ is the activation value of unit i, and n the number of units that send connections to unit j. A bias unit (n=0) is included. The computation of the weighted sum is followed by application of the sigmoid function. The majority of applications with artificial neural networks are computed by 3-layer MLP: this means that each neuron is connected to all neurons in the next layer. Each interconnection has an associated weight, denoted by w, with subscripts that uniquely identify the interconnection. The last layer is the output layer, and activation levels of the neurons in this layer are considered to be the output of the neural network. Thus the general form of Equations 1 and 2 becomes:

a_j,k+1_ = 1/1 + exp (-Σ w_ji,k_ a_i,k_) (3)

in which a_j,k_ represents the activation values of node i in layer k, and w_ji,k_ represents the weight associated with the connection from the i^th^ node of the k^th^ layer to the j^th^ node of layer k+1. Because there typically are 3 layers of nodes, there are 2 layers of weights, and k = 1 or 2 [12].

The weights on all the interconnections are initially set to be small random numbers and the artificial neural network is untrained at this stage. Then the network is presented with a training data set which provides inputs and desired (or known) outputs to the network. This is the stage whereby weights are adjusted in such a way that the likelihood the network will compute the desired output at its output level does increase. Therefore, training means many presentations of data to the neural network and the adjustment (often performed by a gradient descent computation) of its internal weights, until appropriate results are output from the network. This happens by searching a minimum error that can be attained during computations. After these error values are known, weights on the incoming connections to each output neuron then can be updated, which is proportional to a learning rate parameter used to update an error value for each hidden node [12].

It is quite clear that results with artificial neural networks depend on the data with which they are trained, to the extent that these methods are excellent at identifying and learning patterns that are in data. In more general terms, there have to be patterns present in the data before the neural network can learn the patterns successfully. If the data contain no predictive factors, then the neural network performance cannot be high. The informative content present in the data is accordingly a major limiting factor of artificial neural network performance. Under this perspective, these models represent a set of equations, linked together, through shared variables, in a format of interconnected nodes. Equations are not “new”, since they were in existence (like the sigmoid function) before they were labelled “neural networks”. What is important here is that the equations form a system with powerful and far-reaching learning capabilities, whereby complex relations (especially when the inputs are numerous) can be learned during training and recalled later with different data, possessing however a comparable general structure. A network’s diagram, such as the following, in relation to the present study (see Appendix 1 and Table 2 for abbreviations; ALL45 stands for all 45-year all cause deaths to be predicted):


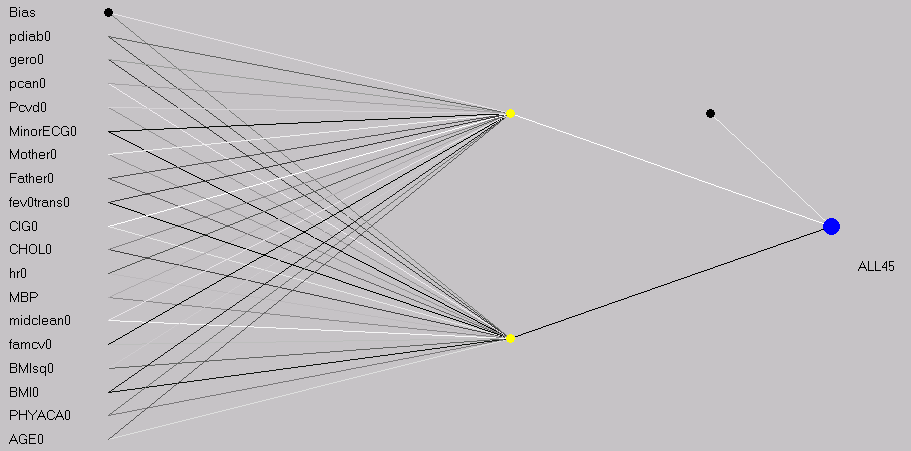


shows how the equations are related, illustrating the inputs, outputs, and desired outputs, an intuitively easier method to conceptualize compared with systems involving equations alone [12].

Although tabulations of weights derived from trained MLP have been occasionally published [28], these weights are not immediately as useful as are regression coefficients in case of standard multivariable methods such as logistic models [29]. This is due to the difficulties of immediate weight re-application and the complexity of formulae needed to calculate risk [28], which is instead mathematically easy with multiple logistic function [1-3,13]. Moreover, in order to interpret multiple logistic function solutions, logit transform enables risk calculations [1-3,13]. With neural networks a cross entropy error function to adjust the weights and to minimize the network fit criterion has been used by Voss et al. [28]. The cross entropy function can be derived from the likelihood of the underlying Bernoulli distribution of the entire training set and it is specially designed for classification problems, in combination with the logistic activation function which maps all its arguments to values between 0 and 1, in the output layer of the network, yet being cumbersome to calculate.

With Tiberius software, model estimate probabilities are calculated versus actual probabilities, which is exemplified in the following graph for MLP neural network:


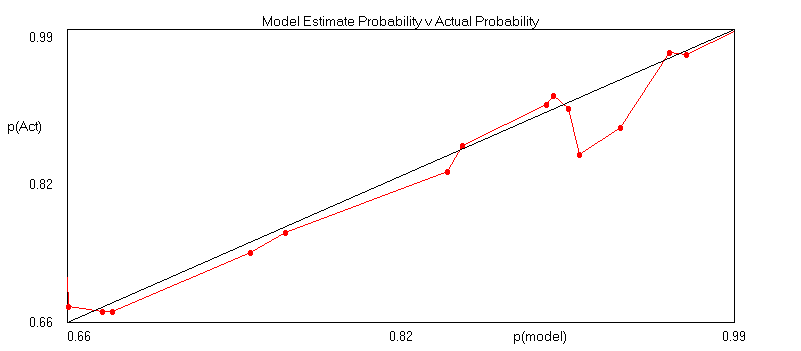


The graph relates to all-cause mortality in 45 years among all 1591 IRA SCS individuals with complete data (for all variables considered) out of the 1712 enrolled at entry.

Weights are not tabulated in the present investigation. We will provide, on request, html files whereby the individual 45-year all-cause mortality risk may be calculated based on neural network models (either forced or stepwise) and risk factors values digitized in the ranges of those measured in the IRA SCS cohort whereby the models were obtained.
